# Prospective observational research on the clinical profile and outcome analysis among a cohort of patients sustaining traumatic cervical spine and cord injury in a peripheral tertiary spine care centre in Nepal

**DOI:** 10.12688/f1000research.12911.1

**Published:** 2017-11-06

**Authors:** Sunil Munakomi, Binod Bhattarai, Iype Cherian

**Affiliations:** 1 Department of Neurosurgery, College of Medical Sciences, Chitwan, Bharatpur, 44207, Nepal

**Keywords:** Trauma, Cervical spine, Spinal cord, Outcome

## Abstract

**Background: **In developing nations like Nepal, spinal cord injury has multispectral consequences for both the patient and their family members. It has the tendency to cripple and handicap the patients, and burn out their caretakers, both physically and mentally. Furthermore, the centralization of health care with only a handful of dedicated rehabilitation centers throughout Nepal further places patients into disarray. This study was carried out as a pilot study to determine the modes of injury, age groups affected, clinical profiles and patterns of injury sustained, as well as the efficacy of managing a subset of patients, who have sustained cervical spine and cord injuries.

**Methods: **This was a prospective cohort study comprising of 163 patients enrolled over a period of three years that were managed in the spine unit of College of Medical Sciences, Bharatpur, Nepal.

**Results: **Road traffic accidents were implicated in 51% of these patients. 65% of them were in the age group of 30-39 years. Traumatic subluxation occurred in 73 patients with maximum involvement of the C4/5 region (28.76%). Good outcome was seen in patients with ASIA ‘C’ and ‘D’ with 55% of patients showed improvement from ‘C’ to ‘D’ and 95% of patients showed improvement from ‘D’ to ‘E’ at 1 year follow up. The overall mortality in the patients undergoing operative interventions was only 1.98%.

**Conclusions:** The prevalence of cervical spine injuries in the outreach area is still significant. The outcome of managing these patients, even in the context of a resource limited setup in a spine unit outside the capital city of a developing nation, can be as equally as effective and efficient compared to the outcome from a well-equipped and dedicated spine unit elsewhere.

## Introduction

Spinal cord injury (SCI) remains one of the most devastating incidents to happen to an individual ^1^. This not only has multispectral negative impacts to the affected individual, but also has an ill effect on the individual’s family members, society and nation as a whole.

The United Nations has recently implemented the “Decade of Action for Road Safety” with an aim of reducing this problem globally ^2^. In Nepal road traffic accidents area major cause of spine injuries. Therefore, globally there are certain reforms being applied to reduce the incidence of SCI, such as the implementations of regular traffic checkups, laws on the use of seat belts while driving and increasing public awareness through media ^3^.

According to a report by the World Health Organization, 82% of the victims with SCI are male, with the majority of them (56%) in the age group of 16–30 years. To make the matter worse, 50–60% of them remain unemployed following the tragedy ^4^. Such injuries have tremendous consequences on the overall resource allocations in many developing nations.

Studies have shown that hospital acquired pneumonia and wound infection propagate disability and mortality in patients with SCI ^5^. Therefore, these complications bear negative impacts on patients’ overall functional outcome and their quality of lives ^6^. Re-admission rates within a year for such patients have been found to be as high as 27.5% ^7^. One cross-sectional study from the US Healthcare System found out that 95.6% of SCI patients had at least one medical complication at the time of their routine annual check-up ^5^.

There has been a recent suggestion of incorporating multifamily group interventions and active educations to improve the overall outlook of SCI patients ^8^. This approach also helps minimize burn out among the care givers who are encountering a new role. Most often, there is only the manpower available for providing necessary care for sustaining critical support for these patients ^8^.

Most patients with SCI have problems achieving a positive outlook and perceiving a sense of self-efficacy ^9^. Confidence or self-efficacy in managing SCI in many community-living people with SCI is suboptimal ^10^. This means that the caretaking aspect becomes an “unexpected career”, and they have to enter this new role without any preparation or specialized training ^11^. There is also “post-injury shift in relationship dynamics” from family members to that of a care provider ^12^. High levels of caregiver burden adds to physical and emotional stress, burnout, fatigue, anger, resentment and depression among caregivers ^13, 14^. Having a community peer support service for individuals with SCI provides psychological and emotional support by a person with a SCI, advice on living with a SCI, practical advice and information, and ongoing support and friendship to the patients and their care-providers as well ^15^.

There are difficulties in managing patients in Nepal due to certain limitations ^16^. The foremost being the poor financial aspects of our people; Nepal has an annual per-capita health expenditure of just $40 ^17^. The next hurdle is that of bureaucracy involved in the custom offices while clearing the ambulances, since the ambulances come from other countries and have to cross international borders. Other hindrances pertain to infrastructure, e.g. road conditions: only 43% of the population has access to all-weather roads, and the inaccessibility of adequate transportation results in delays in providing timely health care ^18^. Logistical (e.g. frequent strikes) and cultural (e.g. public behavior and response to emergency vehicles) problems are also other relevant hurdles.

Qualified professionals are often unwilling to work in low-resource settings given the lack of incentives, thereby there is decentralization of manpower and lack of health facilities outside the capital city ^19^. There is only one truly dedicated spine rehabilitation centre in the whole of Nepal, which is situated in the capital city (Kathmandu). The concept of a peer support group is almost not heard off here. Therefore, SCI patients and their care providers often become neglected, and become separated from society.

This study was carried out to determine the clinical profile of patients presenting with cervical spine and cord injuries at our centre in Nepal (the first centre with a complete armamentarium for managing almost every SCI case scenario outside of the capital city) and also to evaluate the patient’s outcome from the management provided. This study is the first to make a small initial step, thereby motivating others to make a giant leap in decentralizing efficient and effective patient care.

## Methods

This was a prospective observational cohort study of all patients with documented traumatic injury to the cervical spine or its cord, presenting to the Spine Unit at the College of Medical Sciences, Chitwan from March 2013 to March 2016.

### Participants

All patients who presented to our department, either primarily or following their referral from other centers, and were diagnosed of having traumatic cervical spine and cord injuries, were eligible for inclusion in our study. They were enrolled in our cohort study following obtaining their written consent for participation in the study.

Exclusion criteria consisted of any patients with significant poly-trauma or significant medical co-morbidities, those failing to provide written consent for inclusion in the study, patients who left the hospital against medical advice.

### Clinical methodology

**Figure 1.**
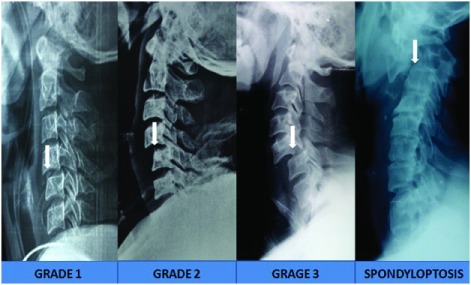
X-ray images showing various grades of traumatic subluxation.

***Imaging of the injury*.** The immobilization of the neck was first secured. National Emergency X-Radiography Utilization Study (NEXUS) criteria ^20^ and Canadian C-spine Rule ^21^ was utilized as guidelines in forming algorithms to obtaining X-ray images ( Figure 1). Further necessary imaging with Computerized Tomography (CT) or Magnetic Resonance Imaging (MRI) of the spine was carried out as and when necessary. CT images helped us in assessing fracture, degree of subluxation and the integrity of the facet joints. MRI images provided us with information on the status of the disc, associated hematomas, degree of compression of the cord, associated cord contusions and the integrity of the posterior ligamentous complex.

***Patient assessment*.** Neurological assessment was first carried out and documented as American Spinal Injury Association (ASIA) grading ^22^. Sensory and motor findings were thoroughly assessed by evaluating single breath count and the presence of Horner’s syndrome was also checked so as to aid in clinical localization. Anal tone and the presence of priapism were also documented. In order to avoid the confounding bias of neurogenic shock, final recordings of the neurological assessment were undertaken 72 hours after the injury, especially in patients with ASIA ‘A’ and ‘B’ grading so as to avoid the confounding bias of spinal shock. In the presence of any deficits, Methylprednisolone was initiated as per the National Acute Spinal Cord Injury Study protocol in all patients presenting within 8 hours of the injury ^23^.

### Mode of management

Further management was undertaken as per the lesions revealed from radio imaging and the clinical assessment of the patients.

**Figure 2.**
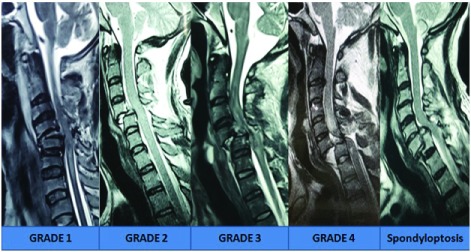
MRI images showing various types of traumatic subluxation, as per the Meyerding grading ^24^.

***Traumatic subluxation*.** In cases of traumatic subluxation, classification was done per Meyerding grading ( Figure 2) ^24^.

**Figure 3.**
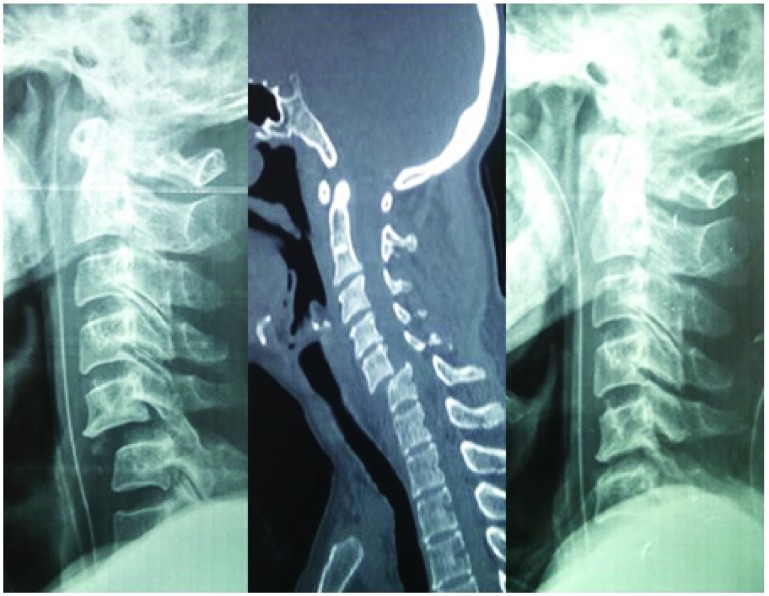
Image showing reduction in the subluxation with realignment of the spine following application of skull traction.

In all cases of Meyerding grade 4 subluxation and spondyloptosis, as well as in cases planned for occipito-cervical fusion and C1 lateral mass screw fixation, CT angiography was also carried out to assess the course of the vertebral artery. Incentive chest spirometric, as well as limb physiotherapy was initiated in all these patients. Guarded traction was applied for reduction in all the patients, with frequent monitoring to prevent over distraction ( Figure 3).

**Figure 4.**
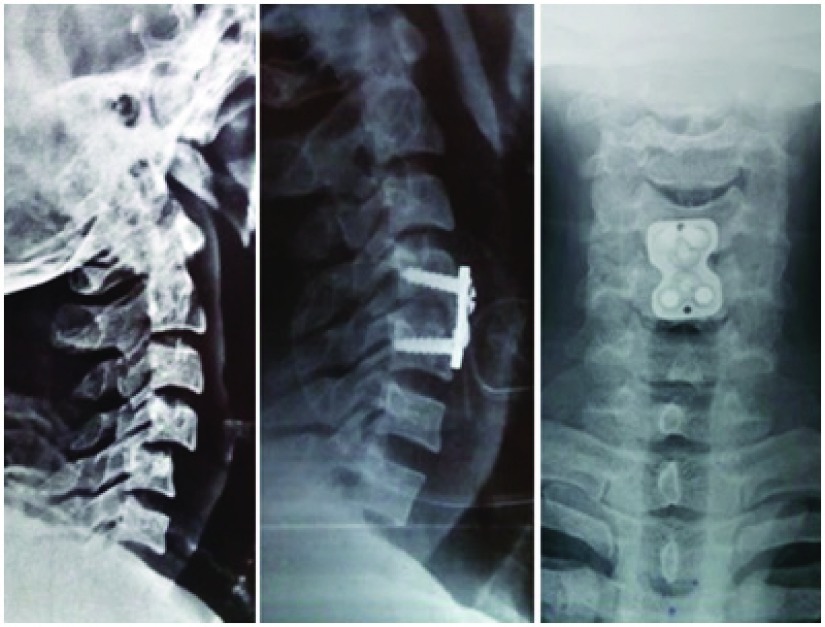
Image showing management in a patient by anterior approach with discectomy and plate with screw fixation only.

If there was good realignment following traction, the patients were managed by either discectomy or median corpectomy followed by *in situ* strut iliac bone graft with plate and screw fixations (anterior cervical approach) ( Figure 4).

**Figure 5.**
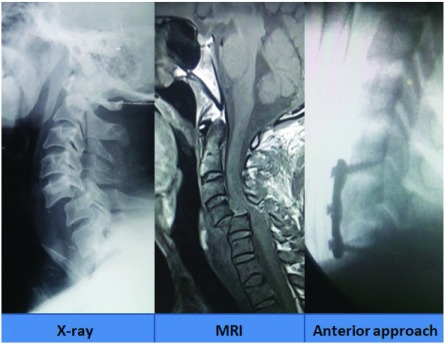
Image of anterior approach only with corpectomy, *in situ* graft placement with plate and screw fixation.

Sometimes, in patients with poor financial status, unassisted bone graft placement was also performed followed by hard cervical collar application for at least 6 weeks. In cases where no reduction was possible with traction (locked facets), then the reduction was tried under anesthesia with muscle relaxants. If reduction was possible, only an anterior approach was taken ( Figure 5).

**Figure 6.**
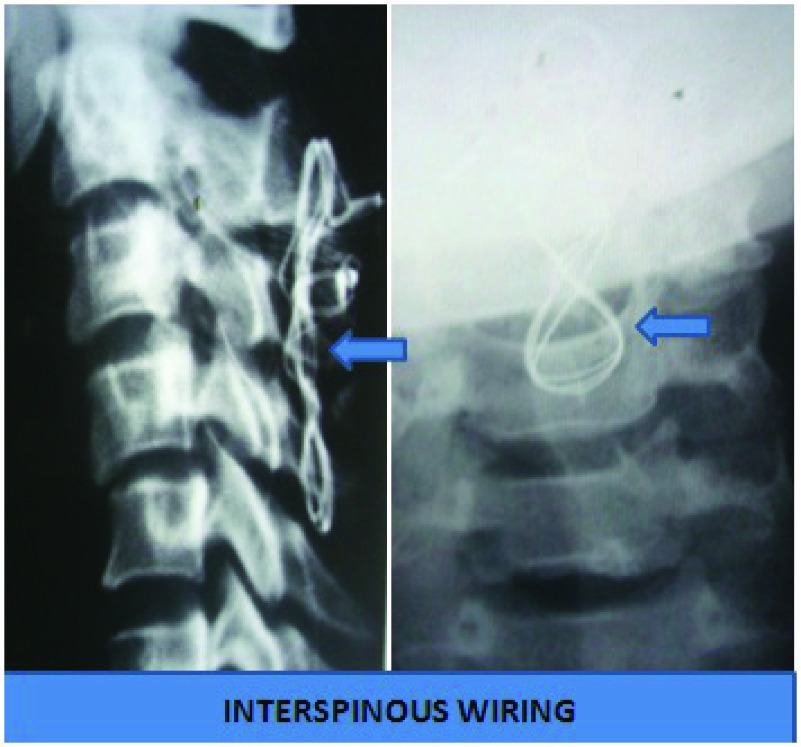
Image of posterior approach with inter-spinous wiring alone.

However, if the reduction was still not possible, then in patients with ASIA ‘A’ and ‘B’ status, only anatomical fixation was ensured by performing the inter-spinous wiring through the posterior approach ( Figure 6).

**Figure 7.**
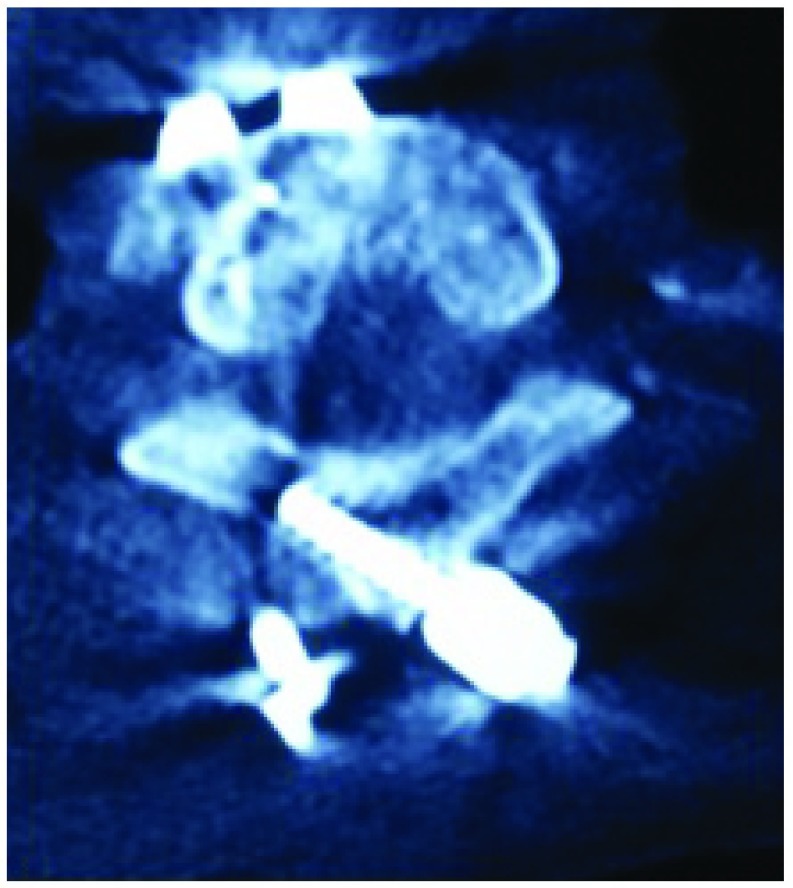
Image showing placement of trans-laminar screw placement.

**Figure 8.**
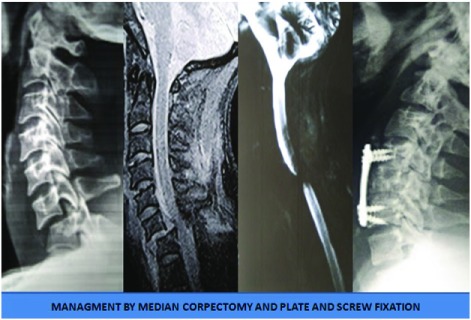
Management by median corpectomy and graft placement with plate and screw fixation.

**Figure 9.**
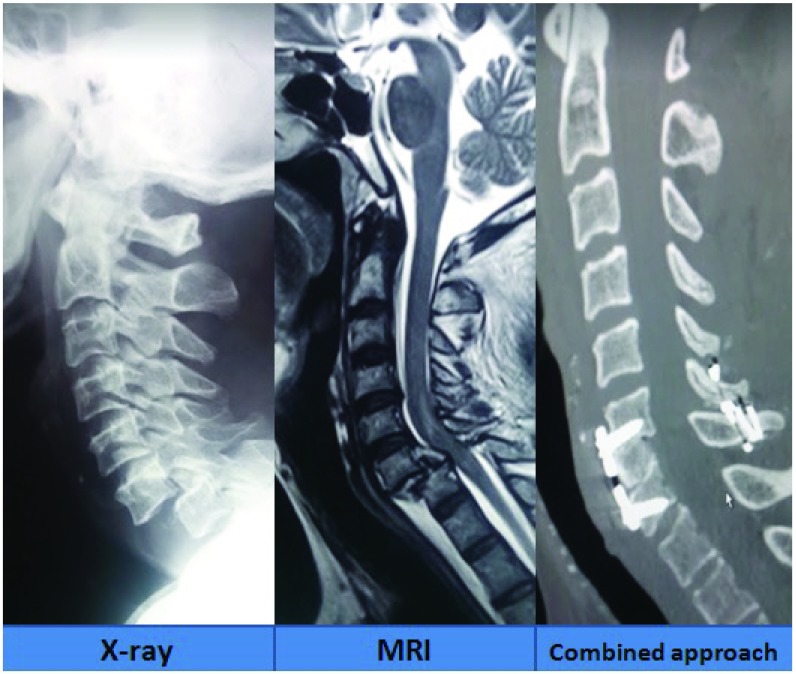
Management in a case with both anterior and posterior approaches.

**Figure 10.**
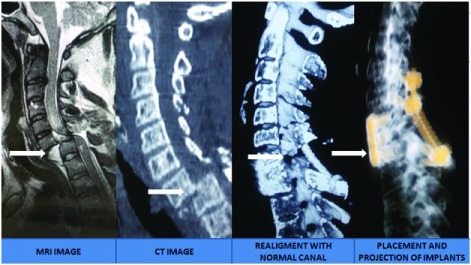
Management in a case of spondyloptosis with no neurological deficits.

The main rational was to allow patients with early mobilization in wheelchairs. In patients with ASIA grade of ‘C’, ’D’ and ‘E’, if there was no significant disc seen in the MRI, then posterior was taken first for unlocking the jammed facets. The posterior instrumentation was then carried out by placement of lateral mass screws with occasional usage of trans-laminar screws and inter-spinous wire placement ( Figure 7). Sometimes, we had to resort to placement of inter-spinous wiring only. This was followed by discectomy or corpectomy and graft with plate and screw fixation from the anterior approach ( Figure 8). However, if there was presence of a significant disc, discectomy or corpectomy was first carried out, then unlocking of the facets with posterior instrumentation was done, followed by placement of the graft with plate and screw fixation from the anterior approach (global approach) ( Figure 9 and Figure 10) ^25^.

**Figure 11.**
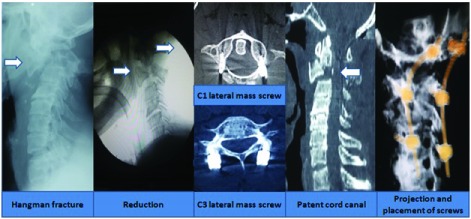
Management in a case of Hangman’s fracture in an 80-year-old male.

***Hangman’s fracture*.** In cases of Hangman’s fracture in young patients, C1 and C3 lateral mass screw and rod placement was undertaken. However, in older patients, above 65 years, occipito-cervical fusion was carried out ( Figure 11) ^26^.

**Figure 12.**
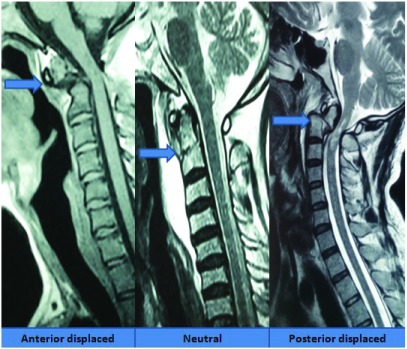
Classification of an odontoid fracture depending on the displacement of the odontoid segment.

**Figure 13.**
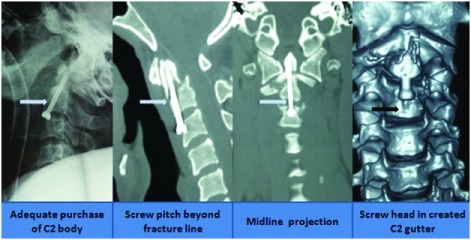
Images showing the basis of performing partial corpectomy in the body while placing odontoid screw.

***Odontoid fracture.*** We classified odontoid fractures into three subgroups depending on the displacement of the fractured odontoid segment in relation to the C2 body: anterior displaced, neutral and posterior displaced ( Figure 12). Realignment was achieved with careful and judicious dorsal or ventral movement of the neck under anesthesia. We have also designed a surgical technique that helps placement of odontoid screws in resource limited settings; with high accuracy ^27^. We create a longus colli gutter so as to completely expose the body of C3. Following a C2-C3 median discectomy, a median gutter is created in the upper body of C3 ( Figure 13).

**Figure 14.**
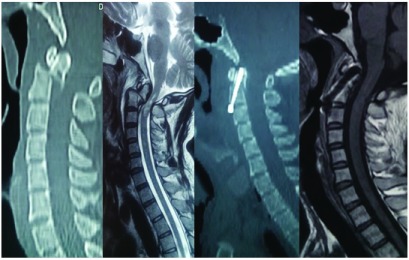
Image showing placement of the odontoid screw and the follow up CT and the MRI scans.

This has many benefits. Firstly, it ensures midline projection of the screw, thereby minimizing the use of C-arm or O-arm, which reduces the risk of excessive radiation hazards. Secondly, it ensures adequate banking of the screw in the cortex of C2, thereby minimized screw pullout. The gutter in C3 homes the head of the screw, thereby minimizing the chances of post-operative discomfort in the patient ( Figure 14).

Whenever possible, MRI compatible titanium plates and screws were used in the surgery (cost, $700). In poorer patients, we opted for alloy implants (cost, $350), and sometimes even steel implants (cost $80).

***Remaining pathologies*.** In patients with central cord syndrome, instability was ruled out by performing dynamic X-ray of the spine. The neck was immobilized in a hard cervical collar. These patients were also started on citicoline (oral tablet, 500 mg three times daily).

All the patients with C1 arch fractures, dear drop fractures and C7 spinous process fracture were stable, and therefore managed conservatively.

### Post-operative management and follow up

All operated patients were aimed for early mobilization in a wheelchair with rigorous chest and limb physiotherapy. The relatives were also taught necessary care protocols ^28^.

All these patients were advised for follow-ups at 2 weeks, 1 month, 3 months, 6 months and then yearly following discharge. Use of telephone interviews and even video calls using social media were used, in order to inquire as to the current status of the patients.

### Data collection

Data gathered about these patients included their clinical profile, ASIA grading, nature and level of their injuries, mode of management, any associated complications and subsequent outcome in their follow up. These data were recorded by residents and were discussed monthly and evaluated by the respective consultants of the Spine Unit. All the patients were prospectively followed up to assess the management undertaken on them and their subsequent outcome in their follow up visits. The records of the patients were stored in the central record store of our hospital and later descriptive analysis was carried out for our study purpose.

## Results

During the study period (March 2013 to March 2016), a total of 163 patients were enrolled and then followed up in our cohort study.

### Patient profile

The age of the patients in our cohort ranged between 2 and 80 years, with 65% of them in the age group 30–39, 19.85% in age group of 40–49, and 17.73% in the age group of 20–29.

**Figure 15.**
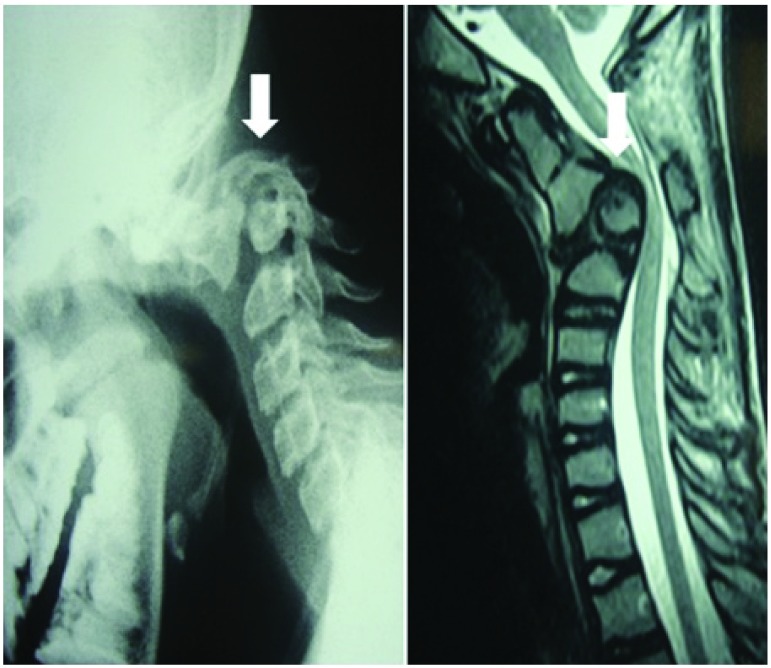
Image showing a case of neglected Hangman’s fracture.

Only 36% of patients had been treated with a cervical collar for neck immobilization at the time of their arrival to the hospital. In addition, only 16% of patients in ASIA ‘D’ or below, presented within 8 hours from the time of injury. One missed, and thereby neglected, case presented 4 years after the injury with a severe swan neck deformity with features of gross myelopathy ( Figure 15).

***Mode of injury*.** Road traffic incidences were implicated in 51% of the cases, followed by fall related incidents for 41% of cases in this cohort group. Minor remaining cases were related to physical assault, playground injuries, animal attacks, earthquake-related incidents and gas explosions.

### Pathology

Traumatic subluxation was seen in 73 patients, followed by odontoid fractures in 24 patients ( Table 1).

**Table 1.** Distribution of various pathologies seen in the cohort group.

Pathology	N
Subluxation	73
Central Cord Syndrome	26
Odontoid fracture	24
Chip fracture	12
Stable body fracture	10
C7 spinous process fracture	8
C1 arch fracture	6
Hangman’s fracture	4
Total	163

A total of 73 patients had traumatic subluxation of cervical spine with maximum involvement in the C4/5 (28.76%) followed by C5/6 (24.65%) region. Most of them had Meyerding Type 1 injury (35.6%) and were in the ASIA ‘D’ neurological status ( Table 2).

**Table 2.** Clinical profile of all the patients with traumatic subluxation of the cervical spine in our study.

CLINICAL PROFILE	ASIA ‘A’	ASIA ‘B’	ASIA ‘C’	ASIA ‘D’	ASIA ‘E’
**Meyerding 1**	C4/5- 1 C5/6- 1 C6/7- 1	C4/5- 1	C4/5- 1 C5/6- 1 C6/7- 1 C7/T1- 1	C2/3- 2 C3/4- 2 C5/6- 5 C6/7- 3	C3/4- 2 C4/5- 2 C5/6- 1 C6/7- 2
**Meyerding 2**	C4/5- 1		C1/2- 1 C3/4- 2 C5/6- 4 C6/7- 1	C4/5- 7 C5/6- 4 C6/7- 1	C2/3- 1
**Meyerding 3**	C5/6- 1		C4/5- 4 C6/7- 3	C6/7- 2	
**Meyerding 4**	C4/5- 2		C2/3- 1 C4/5- 1		C7/T1- 1
**Spondyloptosis**	C4/5- 1 C5/6- 1 C6/7- 1 C7/T1- 2	C5/6- 1	C6/7- 1		C6/7- 1

There were 24 cases of odontoid fractures in our study ( Table 3). Anteriorly displaced variant was seen in 41.66%, neutral type was seen in 41.66% followed by posteriorly displaced variant seen in the rest 16.66% of cases. In patients with central cord syndrome, 60% of them were centered in the C4/5 region, with 58% of these patients presenting in ASIA ‘C’ status.

**Table 3.** Clinical profile of all the patients presenting with odontoid fracture in our study.

S.No	Age/Sex	Mode of injury	Medical Comorbidities	Symptoms	ASIA grading	Associated injuries
1	34/F	RTA	None	Neck pain	E	None
2	30/M	RTA	None	Neck pain	E	Fracture 3 ^rd^ metacarpal bone
3	22/M	RTA	None	UL weakness	C	C4–C5 cord contusion
4	21/M	Fall injury	None	Neck pain	E	None
5	34/M	RTA	None	UL weakness	C	C2–C3 cord contusion
6	15/M	RTA	None	Neck pain	E	None
7	45/M	RTA	None	Neck pain	E	Lung contusion
8	45/M	RTA	None	Neck pain	E	Left Fronto-temporal SDH
9	28/M	RTA	None	Neck pain	E	None
10	45/M	RTA	Diabetes	Neck pain	E	Bladder rupture
11	40/M	Earthquake	None	Neck pain	E	None
12	60/M	Fall injury	Hypertension	Quadriplegia	A	High cord contusion
13	30/M	Fall injury	None	UL weakness	E	C1–C4 cord contusion
14	31/M	Gas Explosion	None	Neck pain	E	Left femur inter- trochanteric fracture
15	55/M	Fall injury	Hypertension	Neck pain	E	None
16	26/M	RTA	None	Neck pain	E	None
17	33/M	RTA	None	Neck pain	E	None
18	28/M	RTA	None	Upper limb weakness	C	None
19	45/M	Fall injury	None	Neck pain	E	Fracture neck of femur
20	39/F	RTA	None	Neck pain	E	Minimal hemo- peritoneum
21	47/M	Fall injury	None	Neck pain	E	Rib fracture
22	19/M	RTA	None	Neck pain	E	None
23	29/M	RTA	None	Neck pain	E	None
24	43/M	RTA	None	Upper limb weakness	E	Radius fracture

### Complications

**Figure 16.**
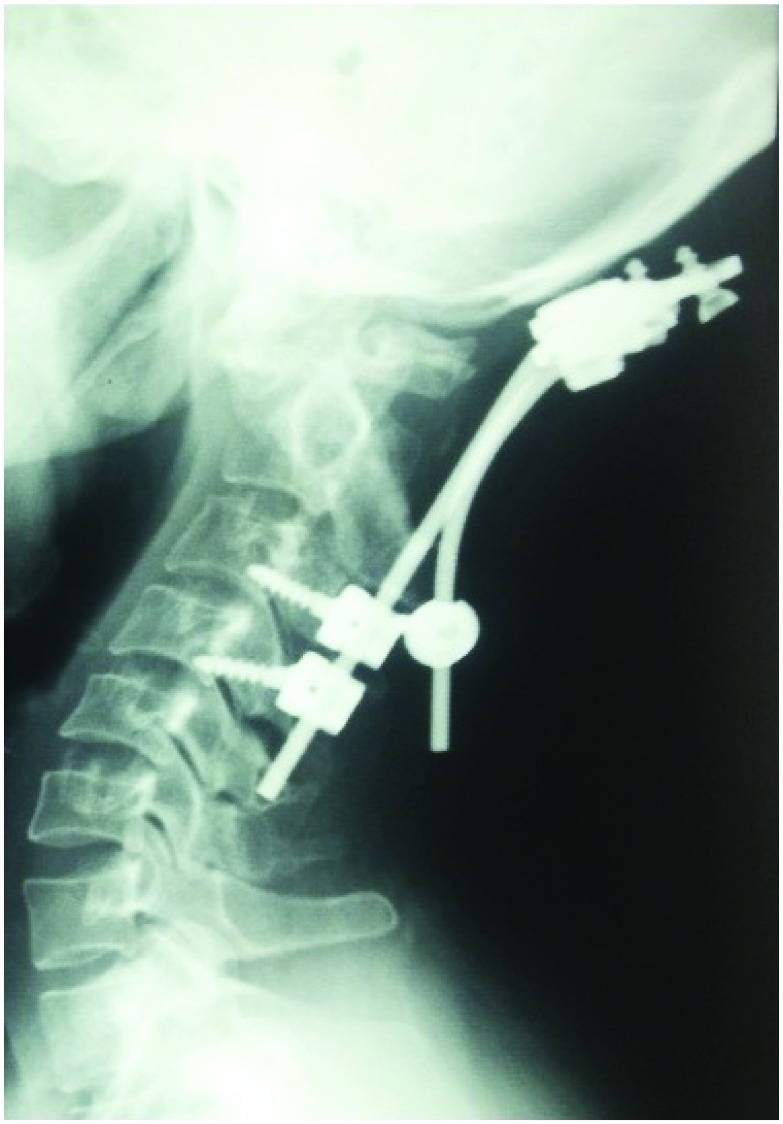
Image showing screw pull-out in the occipital region.

**Figure 17.**
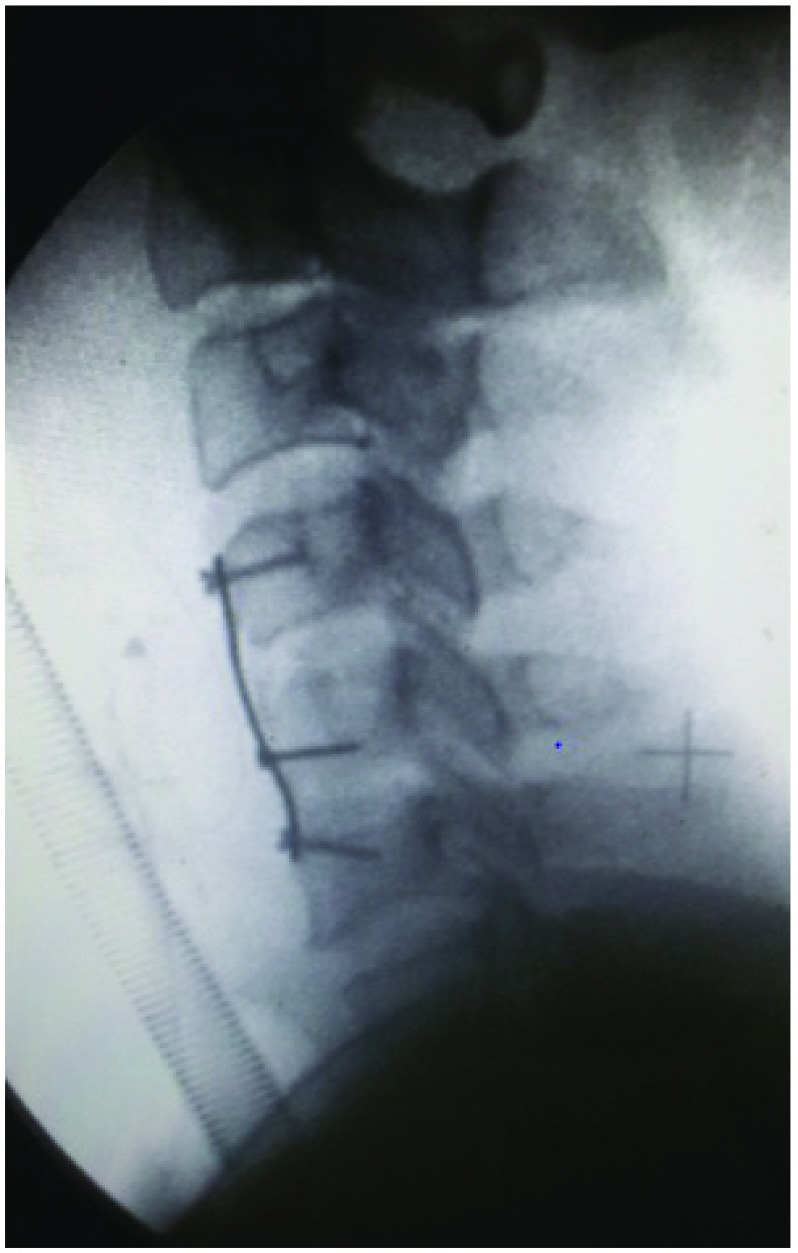
Management in a case of extruded graft by placement of a simple steel plate and screws.

There was 1 screw pullout seen in a case with occipito-cervical fixation in Hangman’s fracture ( Figure 16). The implant was removed after ensuring good fusion at the fracture site. A graft extrusion occurred in a case that underwent an unassisted graft owing to financial restrain. It was managed by replacement of the graft with support from simple steel plate and screws ( Figure 17). One patient had post-operative hematoma in the surgical site requiring its evacuation. Two patients developed superficial surgical site infections, which were both managed conservatively.

**Figure 18.**
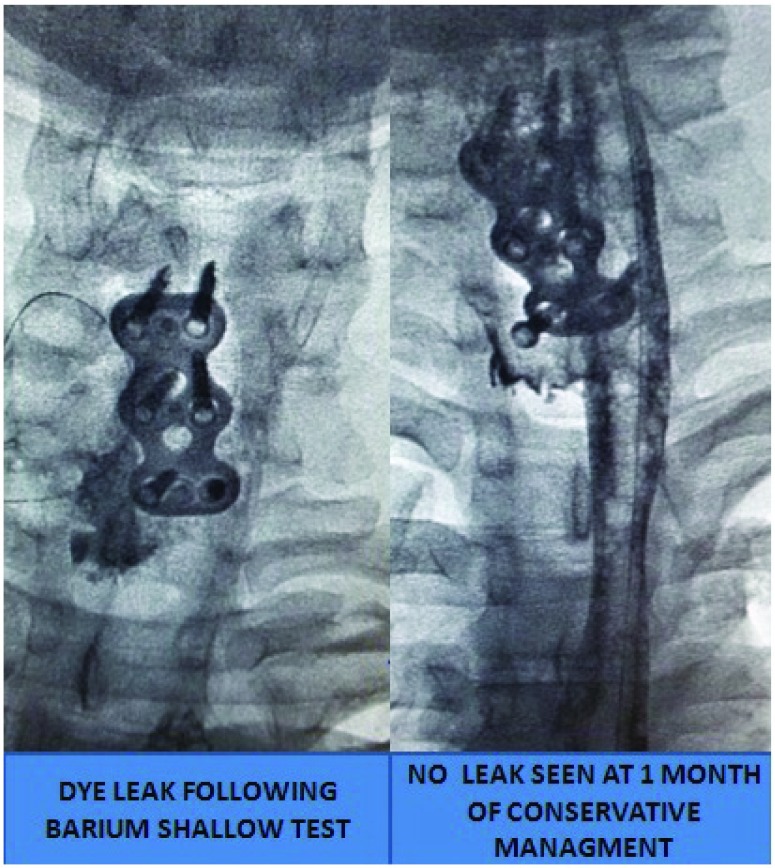
Complete healing of trachea-esophageal fistula with conservative management by placement of naso-gastric feeding tube.

Two patients had trachea-esophageal fistula. One patient was managed conservatively with Ryle’s tube insertion and was healed after a month ( Figure 18). The other patient died of severe mediastinitis despite multiple attempts to repair it.

One patient undergoing odontoid screw fixation had the wrong lateral projection of the screw requiring reinsertion. Another patient with odontoid fracture died following inferior wall myocardial infarction in the fourth post-operative day.

### Recovery

In total, 2 out of 13 patients in ASIA ‘A’ showed improvement to ASIA ‘B’ at 6 months; none of the patients showed improvement from ‘B’ to ‘C’ at 6 months; 55% of the patients showed improvement from ‘C’ to ‘D’ at 1 year; 95% of patients showed improvement from ‘D’ to ‘E’ at 1 year. Only 45% of patients in ASIA ‘A’ and ‘B’ were able to be followed beyond 6 months; 100% of them had developed pressure sores.

Spreadsheet containing the data underlying the results for all 163 patients Click here for additional data file. Copyright: © 2017 Munakomi S et al. 2017 Data associated with the article are available under the terms of the Creative Commons Zero "No rights reserved" data waiver (CC0 1.0 Public domain dedication).

## Discussion

The cervical spine remains the most common level for SCI, representing 55% of all SCIs ^29^. People in the low- and middle-income countries experience 80% of fall related mortality worldwide ^30^.

Without appropriate preventive action road traffic accidents (RTA) are predicted to be the third leading contributor to the global burden of disease and injury by 2020 ^31^. Studies have shown that falls and land transport account for more than 75% of traumatic SCI cases, with almost 30% of them resulting in tetraplegia ^32^.

A prospective observational study conducted in a Tertiary Hospital in India found that RTA caused 62.5% cases, with 21.8% sustaining a C5 level injury ^33^. Another observational study based on autopsy, death due to cervical spinal cord injury, found that men made up 89.4% cases, and young adults (20–39 years) were 63.8% cases. C3-C4 (37.3%) was most commonly involved with 56.6% of the victims dying even before reaching nearby hospitals. The mode of injury was RTA (52.2%) followed by fall from a height (25.0%) ^34^. In our study, 65% of the patients were in the age group of 30–39 years; RTA was the most common cause of injury (51%) followed by fall injury in 41%.

There have been very few studies carried out on traumatic spinal cord injuries in Nepal ^35, 36^. One of these studies reported 149 injuries in the cervical region over a period of three years ^35^. The most commonly involved age group was between 30 and 49 years (44%), with a male to female ratio of 4:1. Fall-related injury was the commonest mode of injury (60%). In addition, 81% of these patients were transported without any neck protection, and the C5 vertebra was the most commonly injured vertebra. In our study, 36% of patients had their neck immobilized with hard collar application. In our study, C4/5 was involved in 28.76% of cases followed by C5/6 in 24.76% of all cases with traumatic subluxation (44.78% of all cases). The same previous study found mostly men were injured with an average age at 40 years, with almost 58% being the sole bread earner being involved in the injury. Another study found that patients presented late for clinical treatment (mean time of almost 40 hours) after the injury ^37^. Only 16% of the patients in our study presented within 8 hours of injury as well. This may be due to us being one of the referral centers for spinal injuries, thereby mostly those selective cases requiring operative interventions were only transferred to us. The remaining cases requiring conservative management would have been managed in other centers as well. This was also true with regards to the ASIA status of the patients presenting to us. Only a few patients with ASIA ‘A’ presented to us, as most of these patients and their relatives were already counseled of the poor prognosis in other centers beforehand and therefore they were not interested in carrying out further treatment. Only patients having some preserved neurological status either in terms of sensory or motor modalities were more likely to seek further expert opinion, and thereby more likely to present to our care center.

Another study from Nepal found 80% of wheelchair users not able to enter their homes independently and 74% of those using mobility aids having maximal difficulties due to physical terrain. 50% of them had no income, and almost half of them did not have easy access to toilet, water source or roads to their home ^38^.

Previous studies have also found that patients with incomplete cervical injuries (Grades C and D) or with edema in MR studies had a better clinical improvement ^39^. This may be the reason for improvement in 80% of the cases with central cord syndrome presenting to our unit, by at least one ASIA score.

In one large series of such patients, surgical mortality was 2.3%, and neurological long-term results were good, with 51% improvement in AIS grade ^40^. The surgical mortality in our cohort study was 1.98%. In our study, 2.5% of patients in ASIA ‘A’, 7.5% of patients in ASIA ‘B’, 55% of patients in ASIA ‘C’ and 95% of patients in ASIA ‘D’ showed neurological improvement.

There are certain fields that need to be monitored in our quest to minimize RTA. The provision for legislation, strict adherence to seat belt use and awareness of safe traffic behaviors can be the stepping stone in this regard ^41, 42^. A study by Dandona *et al*. also noted that enforcing traffic laws, strengthening the driving licensing system, and providing periodic conditioning of vehicles minimized RTA ^43^.

In total, around 16,600 deaths are due to fall-related incidents in Nepal annually ^44^. Awareness among ambulance drivers or even lay persons about the need of immobilization of the neck and back during transportation of patients can prevent many secondary catastrophes. There is now an utmost need to decentralize manpower, and equip centers, as well as providing dedicated spinal rehabilitation centers outside the capital city ^45^.

## Conclusion

In our setting, spinal cord injury has multispectral negative impacts from the patient to society as a whole. The facility for pre-hospital care is not even in its infancy. Furthermore, poor patient transport, e.g. difficult roads, due to the centralization of manpower and treatment centers puts these medical hazards into further disarray. However, small steps in managing patients may prove to be a giant leap in our attempt to provide healthcare management to patients with spinal cord injuries with equally effective therapeutic benefits even in a peripheral set up.

## Ethical statement

This study was cleared by the Ethical Review Board at the College of Medical Sciences, Nepal. Written informed consent was obtained from all patients (sometimes their caregivers provided consent, when patients were not able to do so), regarding their participation in the study and the publication of their relevant clinical data and clinical images.

## Data availability

The data referenced by this article are under copyright with the following copyright statement: Copyright: © 2017 Munakomi S et al.

Data associated with the article are available under the terms of the Creative Commons Zero "No rights reserved" data waiver (CC0 1.0 Public domain dedication). 

Dataset 1: Spreadsheet containing the data underlying the results for all 163 patients. doi, 10.5256/f1000research.12911.d182653 ^46^
