# Supplementary material for: Prospective observational research on the clinical profile and outcome analysis among a cohort of patients sustaining traumatic cervical spine and cord injury in a peripheral tertiary spine care centre in Nepal
Source: F1000Res. 2017 Nov 6;6:1957. [Version 1] doi: 10.12688/f1000research.12911.1 (PMC5701450; doi:10.12688/f1000research.12911.1)
Supplement: Peer review history

# Peer review: Referee response for version 1

**DOI:** 10.5256/f1000research.13997.r27619

**Published:** 2017-11-23

**Authors:** Nikolay Peev

**Affiliations:** 1 Department of Neurosurgery, Salford Royal NHS Foundation Trust, Salford, UK

The paper contributes for the understanding of the problem in a tertiary centre in Nepal. 

 The cervical spinal cord injury is a condition associated with significant mobility and severe invalidity. In that respect a proper understanding and management of the patients suffering with spinal cord injury is of utmost importance. 

 I recommend publishing

I have read this submission. I believe that I have an appropriate level of expertise to confirm that it is of an acceptable scientific standard.

# Peer review: Referee response for version 1

**DOI:** 10.5256/f1000research.13997.r27617

**Published:** 2017-11-13

**Authors:** Juha Hernesniemi

**Affiliations:** 1 Department of Neurosurgery, Helsinki University Hospital (HUH), Helsinki, Finland

This is a nice and important study. I recommend it to be published. My only remarks are the use of percentages: I would use only numbers like 54 per cent/ not 54.54 per cent.

 Otherwise it is ready to be accepted and published!

I have read this submission. I believe that I have an appropriate level of expertise to confirm that it is of an acceptable scientific standard.
